# On the pursuit of the brain network for proto-syntactic learning in non-human primates: conceptual issues and neurobiological hypotheses

**DOI:** 10.1098/rstb.2012.0073

**Published:** 2012-07-19

**Authors:** Christopher I. Petkov, Benjamin Wilson

**Affiliations:** 1 Institute of Neuroscience, and; 2 Centre for Behaviour and Evolution, Newcastle University Medical School, Henry Wellcome Building, Newcastle University, Newcastle upon Tyne NE2 4HH, UK

**Keywords:** language, monkeys, humans, functional magnetic-resonance imaging, hypotheses

## Abstract

Songbirds have become impressive neurobiological models for aspects of human verbal communication because they learn to sequence their song elements, analogous, in some ways, to how humans learn to produce spoken sequences with syntactic structure. However, mammals such as non-human primates are considered to be at best limited-vocal learners and not able to sequence their vocalizations, although some of these animals can learn certain ‘artificial grammar’ sequences. Thus, conceptual issues have slowed the progress in exploring potential neurobiological homologues to language-related processes in species that are taxonomically closely related to humans. We consider some of the conceptual issues impeding a pursuit of, as we define them, ‘proto-syntactic’ capabilities and their neuronal substrates in non-human animals. We also discuss ways to better bridge comparative behavioural and neurobiological data between humans and other animals. Finally, we propose guiding neurobiological hypotheses with which we aim to facilitate the future testing of the level of correspondence between the human brain network for syntactic-learning and related neurobiological networks present in other primates. Insights from the study of non-human primates and other mammals are likely to complement those being obtained in birds to further our knowledge of the human language-related network at the cellular level.

## 1. Introduction

If you can find a path with no obstacles, it probably doesn't lead anywhere.

Frank A. ‘Parson’ Clark, *ca*. 1963

The path towards understanding the behavioural abilities and neuronal substrates that are evolutionarily related to those that humans use for language has been as challenging as it has been informative. Recently, we have seen considerable advances in modern language theory [1–4] and in our understanding of language-related processes (for recent reviews on the neurobiology of syntax, see: Bickerton & Szathmary [5]). Concurrently, work in non-human animals has seen the development of theoretical frameworks on the evolutionary origins of language-related processes [1,6–9]. This has led to an increase in comparative animal studies on ‘artificial grammar learning’ (AGL) [10–12]. As we consider below, AGL paradigms aim to tap into the computational abilities that humans use to learn syntactically structured sequences [9,13,14]. Moreover, songbirds have recently become an important neurobiological model system, in part, because they learn their vocalizations and because their song production seems to reveal ‘syntactic-like’ abilities that are in some ways related to how humans learn to produce language with syntactic structure [6,15]. These are all exciting developments, but, arguably, one area that remains relatively underdeveloped is in advancing mammalian model systems that can provide insights on the cellular mechanisms that might be homologous to those that the human brain uses to support language-related processes. In particular, additional comparative work with non-human primates, although faced with considerable challenges as we consider in this paper, is needed to inform us on the evolutionary changes that are likely to have occurred within the primate order as language evolved in humans [8]. Interdisciplinary efforts will remain important for advancing future treatments for communication and language disorders, and it is likely that major advances will be difficult to achieve if research efforts are limited to the study of select animal species or to the non-invasive approaches that are normally available for studying humans.^1^

In this paper, we focus on the conceptual and technical challenges that are faced in pursuing evolutionarily homologues to human syntactic-learning in mammals such as non-human primates. We provide a description of what we define here as ‘proto-syntactic’ processes and how we might go about studying these behaviourally and neurobiologically and in ways that can facilitate comparative testing with humans and other animals. We conclude by reviewing recent perspectives on the structure and function of the human brain network for syntactic processes, and propose several neurobiological hypotheses that consider the possible combinations of behavioural sequencing capabilities and neurobiological substrates with which different non-human primate species might present.

## 2. A conceptual framework for the pursuit of proto-syntactic capabilities and processes in non-human animals

Syntax can be defined as the ability to learn and to produce grammatical relations between words and word parts in a sentence. However, syntax is not simply the linear sequencing of words (i.e. evaluating the word-by-word relationships between elements in a string). Although we speak and write word-by-word, modern linguistic theory emphasizes that beneath the surface-level of word sequences is an underlying structure, such as hierarchically nested phrases and ‘movement’ (perceived or actual) of syntactic constituents [1,2,5,6,17]. In this section, we consider: (i) two examples of operational definitions of syntactic abilities that could be comparatively studied with non-human animals; (ii) the important distinction between production and learning, the latter of which allows us to ask questions about the learning abilities of animals, which might be better than their vocal production capabilities; and (iii) the idea of an *evolutionary gradient in syntactic complexity* to help us to understand how human syntactic abilities may have evolved from simpler systems. In this regard, we define *proto-syntactic* abilities as those that reflect an evolutionary increase in computational processing capabilities, which comparative testing might reveal to have formed an evolutionary basis for human syntactic abilities.

### (a) A place to start: creating operational definitions of syntactic abilities for comparative testing

Most definitions of syntax reflect capabilities that are uniquely human, such as the ability to learn to produce and evaluate considerable levels of complexity in the hierarchical structure of sentences. Since no other animals have syntax, grammar, words, sentences, semantics, etc. as they are defined for human language, the first major hurdle for comparative study is to be as clear as possible about the operational definition of the *core aspects of some of these abilities* that one hopes to study with other animals. Each operational definition will suggest and constrain the ways in which these abilities can be comparatively studied and with which species these could be realistically explored.

As one example, we might be interested in studying a general aspect of syntactic sequencing ability, operationally defined as follows: An aspect of syntactic structure building is present in animals that can *learn to produce structural relationships between their individual vocalizations* (what we might call ‘syntactic-like’ ability). The italicized phrase, however, suggests that we would need to study species of animals that are vocal learners *and* have communication systems that allow them to combine several of their vocalizations in some sort of a sequence for production. Syntactic-like abilities in non-human animals seem to be closely associated with vocal imitation and vocal learning, such as when songbirds and humpback whales learn to structure their songs. The few animal species known to be vocal learners (humans, songbirds, parrots, hummingbirds, bats, elephants, pinnipeds and cetaceans, [8,15,18–22]) have varying degrees of syntactic-like capabilities. Of these groups of animals, not all are being neurobiologically studied. Thus, some groups of songbirds have become representative neurobiological animal model systems for vocal production learning and syntactic-like abilities. Moreover, although different songbirds show varying levels of song complexity, the structure of their songs are typically described as exhibiting ‘phonological syntax’ [5,23], where different sequencing combinations of the units do not produce different meanings (referred to as ‘semantically compositional syntax’ in humans).

Many mammals, non-human primates included, have a call-based system for vocal communication that lacks the sequencing abilities of songbirds or cetaceans. Most non-human primates are generally thought to produce unitary calls from a limited set of innate or genetically regulated vocalizations, although this perspective is changing somewhat. Recently, Snowdon [24] reviewed the evidence for vocal learning in non-human primates, stating: ‘None of these new results suggest that primates will soon challenge songbirds for vocal virtuosity, but nonetheless the accumulation of results suggests a much greater degree of vocal control and flexibility of production than previously thought.’, also see [8,25]. Moreover, some species of guenons (Old World monkeys) appear to combine their calls into different context-specific call combinations [26,27]. However, as with songbirds, these call combinations lack semantic compositionality [1,2].

Therefore, an operational definition, such as the following, is required to help us to remain empirically grounded regarding the limited vocal production learning and sequencing capabilities of non-human primates: A core aspect of the human syntactic capacity, to learn how sensory elements are appropriately sequenced, might exist in mammals that are able to evaluate whether sequences of auditory or visual elements violate a previously learned structure. This operational definition differs from the one for vocal learners above in two key respects. First, it does not depend on the vocal production capabilities of the animals, which theoretical papers on language evolution have suggested is not necessary [7]. Second, it draws a distinction between learning and production, suggesting that some animals might be able to learn sequences of sensory elements better than they are able to (re)produce them. We evaluate the basis for this claim next.

### (b) The distinction between vocal production learning and auditory learning

It is well known that human receptive capabilities can outstrip productive capabilities. Any learner of a second language will be familiar with the feeling that their ability to understand that language exceeds their ability to produce well-formed sentences in it, and we know that infants are sensitive to certain properties of their native language before they can use them [28]. A related distinction is made between ‘auditory learning’ and ‘vocal production’ by comparative scientists because many vertebrates are capable of some form of auditory learning although very few species are also production vocal learners [19]. Linguists tend to focus on receptive abilities when they evaluate human language—particularly the ability to differentiate between well-formed and ill-formed (ungrammatical) sentences. However, when scientists look for correspondence to abilities in other animals there is a strong tendency to focus on production [1], such as syntactic-like abilities in songbirds [6,15]. Although many vertebrates are often considered to be vocal non-learners, many of these animals are capable of considerable auditory learning [25,29]. Thereby, the extent to which different animal species can learn varying levels of complexity in how sensory elements are temporally sequenced remains an open question and is an issue that remains linguistically relevant.

### (c) The notion of an evolutionary gradient of syntactic complexity

**Figure 1.**
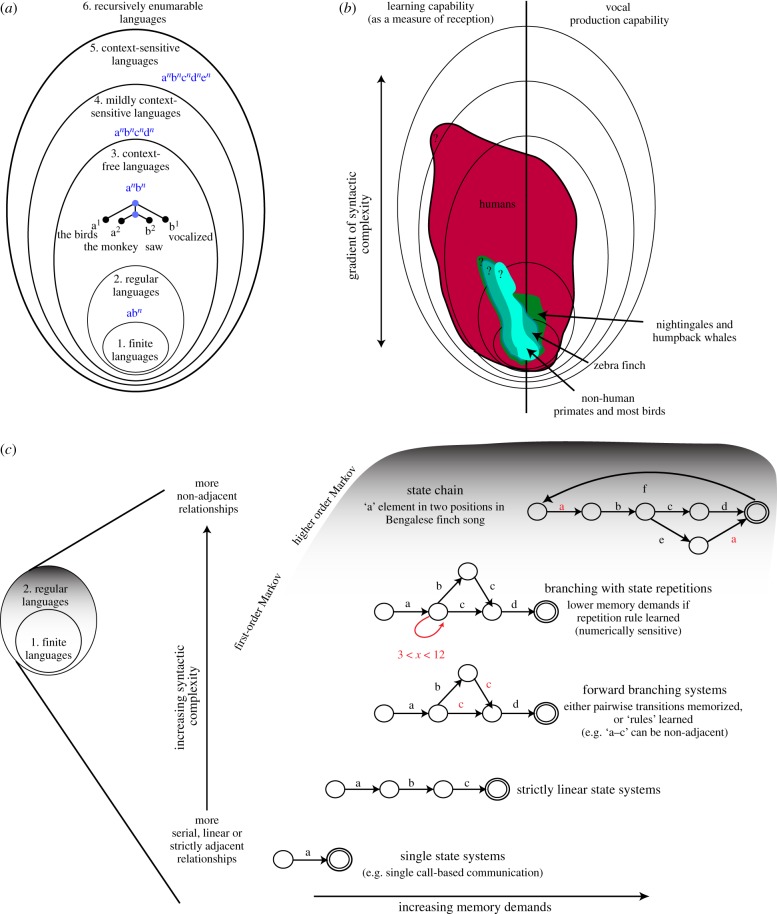
Formal language hierarchy (FLH) distinguishing learning versus production and the notion of a gradient of syntactic complexity. (*a*) Schematic of FLH, based on Berwick *et al*. [6]. (*b*) Our illustrated distinction between learning and production in relation to the FLH, highlighting considerable uncertainty in how high human and other animal learning (rather than production) capabilities can reach in the hierarchy, see text. (*c*) Schematized for quantifying the different dimensions of syntactic complexity, on the vertical axis, a measure of linearity can be used as a function of increasing memory demands on the horizontal axis. Other ways of quantifying complexity in different dimensions would also be useful to test. At the lowest level are single element/state systems, followed by multi-state linear systems with (i) only ‘adjacent relationships’, (ii) forward branching systems including ‘non-adjacent relationships’ that some animals might be able to learn, and (iii) state repetitions that might tap into numerosity sensitivity. Higher still are ‘state chains’ that cannot be solved by first-order Markov models; here the transition following each ‘a’ element depends on the preceding state transition. This process is second-order Markov, but all other transitions are first-order Markov processes [1].

The formal language hierarchy (FLH; or extended Chomsky hierarchy [4]) contains several categories of grammar, each describing an increasingly powerful computational language (see figure 1*a*, which is based on Berwick *et al*. [6]). Here, lower ranked grammars (e.g. finite-state grammars (FSGs); also referred to as ‘sub-regular’ grammars [4]) generate sets of languages that are subsets of the sets of languages generated by higher ranked grammars. Humans seem to be unique in the animal kingdom in being able to produce languages that breach into the realm of context-sensitive languages [30] (figure 1*a*). However, as Hurford notes: ‘ … linguists pay little attention to classes of languages of [the] lowly rank on the Formal Language Hierarchy.’ [1]. In our view, this has resulted in a lack of resolution of the level of complexity of FSGs that are not human unique, leading to an emphasis on determining whether the status of some non-human animal species can be elevated if they are able to learn context-free patterns from context-free grammars (CFGs; also referred to as ‘supra-regular’ grammars [3,4,31]). Moreover, the interpretation that songbirds can learn CFG [12,32] has been questioned for several reasons considered in detail elsewhere [6,33,34], leading Berwick *et al*. [6] to conclude that: ‘Considerable controversy remains as to whether any nonhuman species can truly recognize strictly context-free patterns’. Context-free pattern learning may someday be demonstrated in certain animals [4], yet, even if it is not, it remains important to understand how human capabilities with CFGs and beyond may have evolved from abilities lower in the FLH that are present in other living animals. This requires better resolution of the lower parts of the hierarchy (figure 1) and consideration of the distinction between learning—as a behavioural measure of reception—and production. As we schematize in figure 1*b* for humans and other species of animals, these two behavioural phonotypes should be distinguished (see [25]).

How might the ability to generate context-free languages or beyond have evolved? One possibility is that when the ancestors to living humans began to organize vocalizations and then words into sentences of greater complexity, this built upon the evolutionarily conserved ability to process sets of serially ordered strings. Then at some point selective pressures to reduce memory demands may have expanded syntactic capabilities by the adoption of rule-based learning strategies that avoid having to memorize all the elements and transitions in the sequences from more complex grammars [35]. In this regard, we are motivated by Hurford's attempts to resolve several of the stages below CFGs in reference to the various levels of complexity seen in the songs of songbirds and humpback whales [1]. We will expand on some of his ideas to illustrate our proposed notion of an *evolutionary gradient of syntactic complexity*. See Jäger & Rogers [4] for other approaches to resolve the sub-regular grammar space in the extended Chomsky hierarchy.

One of the simplest scenarios is for a system to recognize and/or to generate single elements. Such is the case for animals with call-based systems that can produce and recognize single vocalizations from a limited set of vocalizations (figure 1*c*). The next level of sequencing complexity is introduced when two calls are combined where it then becomes important to evaluate the ‘adjacent relationships’ between element pairs. The subsequent level of complexity occurs when several elements are serially sequenced in a purely linear fashion. An example of this is the linear song components of, for example, zebra finch songs [36], where the pairwise transitions can be modelled by a first-order Markov process [1] (figure 1*c*). Adding more elements or transitions does not change the computational complexity of the pairwise sequencing process, but requires a larger indexical memory store. Some songbirds, such as Bengalese finches, nightingales and chaffinches, and humpback whales have songs that show sequencing elaborations such as forward or backward branching relationships and elaborations such as repeating elements within a range of acceptable repetitions. While it is not always clear which of these would be hierarchically higher than the others in terms of syntactic complexity (however defined), these sorts of transitions deviate from strictly linear processes [37], although these cases still only require first-order Markov processes to model them (figure 1*c*). Of special interest are the branching transitions since these can be modelled either as a number of adjacent relationships, or could include more complex ‘non-adjacent relationships’ where an optional element can occur between two other elements with some probability. The recognition of non-adjacent relationships can reduce the need to memorize many pairwise transitions if the non-adjacency ‘rule’ can be learned. For adult humans, non-adjacent relationships can include even greater levels of complexity (e.g. nested or crossed relationships [38,39]). Moreover, the ability to deal with non-adjacent relationships is not present at birth but seems to occur during infant development [40,41]. As a final example of another level of syntactic complexity (figure 1*c*), Hurford notes the special case of the same element occurring in multiple parts of the sequence where its next transition state depends on the preceding, called a ‘state chain’ process [1]. Such transitions require higher order Markov models, although much of the rest of the sequence could remain a first-order Markov process.

We hope that these examples help to illustrate the great variety seen in animal song production that can be usefully applied towards quantifying the structural complexity between different artificial grammars, prior to using these in comparative tests with different animal species. It would be of benefit to many if the scientific community works together to rank the complexity of these structures along different dimensions (using quantitative rather than qualitative descriptions, wherever possible). Subsequently, the learning abilities of animals can be evaluated along the various dimensions of ‘syntactic complexity’ to advance our understanding of the evolutionary bases for human syntactic abilities. It remains possible that the evolution of syntactic complexity may have been step-wise rather than, as we have proposed, a gradient function. Yet, if the pursuit is informative regarding how language may have evolved, we welcome the testing of different alternative hypotheses. As we will discuss in §4, there is already a basis for considering syntactic complexity from the human cognitive neuroscience literature, where, for instance, the comparison of adjacent versus non-adjacent relationships (broadly defined) seem to be able to predict which parts of the human language network are engaged [42].

## 3. Obtaining comparative data on artificial grammar learning: implicit versus explicit learning

Classically, the behavioural approach has been a tool of choice for comparative biologists and psychologists. However, even behavioural testing is challenging to apply in the same way across species that may have different forms of communication, different levels of motivation, varying abilities to engage in behavioural testing and that may find different methods of providing responses more natural than others. Combining behavioural study with neurobiological measurements that can be performed in a similar way across the species escalates the obstacles to success. Yet, bridging techniques and approaches are required to link research based on the study of different species. In this section, we consider (i) how AGL can be used to study implicit or explicit learning processes and (ii) several approaches in which behavioural and neurobiological data can be similarly obtained across species to facilitate comparative testing.

The use of AGL paradigms is a promising approach for understanding what aspects of syntactic-related patterns can be learned by animals. Following Chomsky's theoretical formulations of the structure of language [17], Reber pioneered the use of artificial language paradigms to study how humans learn language structure [10]. AGL paradigms have been used to explore the types of structures that humans (including infants and adults), songbirds, non-human primates and rodents can learn [33,43–47]. However, there are differences in how some of these study groups have been tested such that different learning substrates might have been engaged.

The infant and non-human primate data have tended to be obtained relying on the implicit learning of artificial grammars, which is often studied by measuring preferential looking during habituation/dishabituation paradigms [11,14,44,48]. Typically, these experiments are conducted by familiarizing the individual for some length of time with exemplary sequences of stimuli that follow the artificial-grammar pattern or rule(s) [11,12,14,33,44,45,48]. Then in the second ‘testing phase’ of the experiment, the individual is tested with well-formed ‘correct’ or ‘violation’ sequences during natural response measurements, such as preferential looking towards the audio speaker that presented the test sequence. In this way, the familiarization and testing need not engage perceptual awareness for learning to have occurred, i.e. implicit learning [11,44,45,48]. However, in the bird and rodent studies, the participants were trained to discriminate correct versus violation sequences, which could engage an explicit rather than implicit learning system [12,33,46,49]. Similarly, in many of the human studies [43,50,51] either during the familiarization phase or during the testing phase, the participants were engaged in learning the sequencing structure of the artificial grammar by being asked to judge whether the sequences were correct or violation sequences. When participants are actively seeking to determine the artificial-grammar pattern, there is a risk that they might fail to learn some of the sequencing relationships after the point at which they feel that they have sufficiently understood the pattern and are performing reasonably well. Such explicit learning could engage different brain circuits [52] in relation to studies of AGL using implicit learning (such as those in infants and non-human primates).

More recently, the groups of Hagoort and Petersson have worked to engage adult humans in more implicit learning paradigms, whereby little instruction is given to participants during testing other than to report by pressing one of two buttons their preference for a test sequence (i.e. whether they ‘liked’ the sequence or not). Subsequent to this, the participants were asked to make ‘grammaticality’ judgements both to validate the preference judgements and to engage explicit learning [38,39]. Interestingly, both implicit and explicit AGL is reported as yielding fairly comparable results. Both seem to engage the inferior-frontal gyrus (IFG), e.g. Broca's territory (Brodmann areas (BA) 44/45), as has been reported in several other human AGL or natural language-learning studies.

When comparing data with animals such as non-human primates that are limited vocal learners, an advantage of using implicit rather than explicit learning of artificial-grammar sequences is to avoid engaging the aspects of the network that in vocal learners such as humans and songbirds might form part of the network engaged in vocal production. Implicit learning might be better able to distinguish perception from *motor production in the service of perception* by reducing the ability of vocal learners to rely on sub-articulation, imitation, etc. to assist in the perception of syntactic sequences. Otherwise, several aspects of the networks that support syntactic or syntactic-like learning in vocal learners would by comparison to vocal non-learners appear to be strikingly different (e.g. human or songbird unique). For a more detailed discussion of the similarities and differences in the behaviour and neurobiology of vocal learners (such as songbirds and humans) and other animals with more limited vocal learning abilities (such as non-human primates and other birds), see Petkov & Jarvis [25].

**Figure 2.**
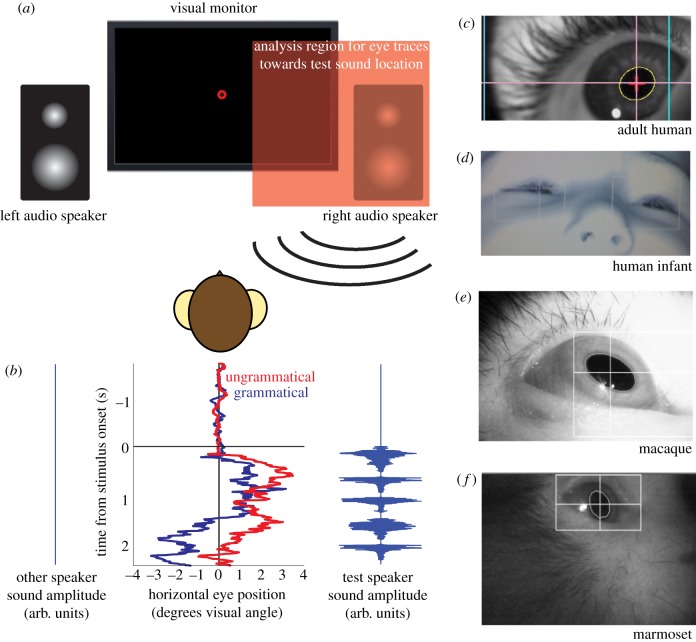
Eye-tracking measurement of implicit artificial grammar learning. (*a*) Schematic of a behavioural eye-tracking experiment with monkeys in our laboratory. The monkey sits in front of a monitor and after a brief central fixation period, an auditory test sequence is randomly presented from the left or right audio speaker. The length of time spent looking into the predefined analysis region around the presenting audio speaker is measured. (*b*) Exemplary eye-traces towards correct (‘grammatical’) and violation (‘ungrammatical’) sequences. Positive values in the plot indicate eye movements towards the test speaker location, whichever audio speaker it was; negative values are looks in the opposite direction away from the presenting audio speaker. (*c*–*f*) exemplary non-invasive infra-red eye tracking of (*c*) adult human, (*d*) human infant (image courtesy of J. Read), (*e*) macaque and (*f*) marmoset.

Another way in which comparative testing can be facilitated is to use similar behavioural and neurobiological measurements between humans, infants and non-human animals. For instance, for behavioural testing, infra-red eye tracking has become more available in scientific laboratories and can be used to evaluate preferential looking responses after habituation to artificial-grammar sequences. This is shown for macaques in figure 2*a*,*b* and can be comparably conducted in adult humans, infants and other types of monkeys, such as marmosets (figure 2*c*–*f*). Apart from the advantage of using eye tracking to measure implicit learning similarly across participant groups, the approach also offers a more objective way to analyse behavioural data, in relation to the traditional approach of manually rating the animals’ responses as captured on video, which has been criticized [34]. Other groups have opted to use brain potentials both to obtain neurobiological data after AGL and to evaluate whether, for instance, infant brain potentials show a signature of learning [40].

Many neuroscientific studies are conducted in anaesthetized animals. However, comparative AGL studies using evoked potentials or brain neuroimaging will depend on the animals being studied awake, rather than anaesthetized. Technical advances have made it possible to accommodate non-human animals so that they can be scanned awake with functional magnetic-resonance imaging (fMRI), which is often used to scan humans [53,54]. Moreover, although the gradient systems of MRI scanners generate a considerable amount of noise, animal MRI studies often use strategies to reduce the impact of scanner noise on the animals and to improve the auditory activity response during sound stimulation [55,56]. Recent fMRI and positron emission tomography (PET) studies have been describing how the brains of non-human primates process communication signals (monkeys [57–60]; chimpanzees [61]). General summaries are now available on how the results in monkeys and apes relate to how the human brain processes species-specific communication signals [62,63]. In this way, testing for the level of correspondence across the species, rather than assuming that it exists, provides a stronger bridge between human neuroimaging work and studies in certain species of non-human primates, where for instance the processing of communication signals can be studied at the neuronal level [64–66]. As a specific example, an fMRI-based correspondence has recently been suggested between how human [67,68] and monkey [57] brains process voice content in communication sounds; see Petkov *et al*. [62]. Subsequently, fMRI-guided electrophysiology was used in the monkeys to target fMRI-identified voice-sensitive brain clusters which when studied seemed to reveal ‘voice cells’ in the primate brain [69]. A similar two-stage approach—linking human neuroimaging results on language-related processes using a bridging technique followed by the neuronal-level study of potential homologues in an animal model system—could provide novel insights into the cellular function of evolutionarily conserved regions than in humans evolved to support language-related processes.

## 4. Neurobiological hypotheses on the proto-syntactic learning network in monkeys

There is a growing consensus among scientists that the prominent brain regions in humans that are engaged in syntactic processes involve the left inferior and middle frontal cortex, large parts of the superior and middle temporal cortex, parts of the parietal cortex and subcortical regions such as the basal ganglia, as well as a number of these same regions in the right hemisphere [42,70]. Many of these brain regions appear to be engaged both during syntactic processing of natural language [71,72] and when human participants evaluate artificial-grammar sequences [13,38,43,50]. Thus, a considerable amount of language-related processing does not appear to be strictly language-specific. Friederici [42] has recently proposed an extensive model integrating information on the structure, function and connectivity of the human brain network that subserves language processing. Important to this model is how different behavioural demands can engage different aspects of the language network [42], thus, we next overview some of the key concepts that are relevant for neurobiological hypotheses of proto-syntactic networks in non-human primates. For other models, including those that focus on human speech processing and the relevance to brain pathways for auditory processing in primates, see [73,74].

*— Several language pathways.* Human semantic and syntactic processing engages several brain pathways: two dorsal pathways link posterior temporal and parietal lobe regions with either premotor cortex BA 6 (dorsal pathway I; via the superior longitudinal fasciculus (SLF)) or BA 44 in Broca's territory (dorsal pathway II; via the arcuate fasciculus, a part of the SLF). Two ventral pathways are hypothesized to link anterior supra-temporal lobe regions and either BA 45 in Broca's territory (ventral pathway I; via the extreme capsule (EC) fibre system) or the frontal operculum (FOP) area below BA 44/45 (ventral pathway II; via the uncinate faciculus (UF)).
*— Syntactic complexity demands on the network.* For initial syntactic structural analysis, the FOP and ventral pathway II are engaged (including for finite-state grammars such as (AB)*^n^* that monkeys and songbirds appear able to learn [11,12,50]). Dorsal pathway II (arcuate fasciculus) and BA 44 are critical for syntactic function, such as evaluating hierarchical structure and ‘non-adjacent relationships’ of various types [13,43]. Dorsal pathway II (to BA 44) and ventral pathway I (to BA 45) are engaged in semantic and syntactic relationships or syntactic movement (e.g. evaluating whether a sentence structure is subject–verb–object versus object–subject–verb). Higher memory demands and longer distance non-adjacent relationships engage Broca's territory (BA 44 in particular) *and* dorsal pathway I to premotor cortex. However, dorsal pathway I is primarily involved in sensory-to-motor mapping.
*— Left hemisphere dominant and subcortical structures can be engaged.* The syntactic/semantic network in frontal cortex tends to be left lateralized, see also [72,75]. The right hemisphere is thought to mainly subserve functions such as the prosodic and emotional aspects associated with linguistic comprehension. Subcortical structures such as the hippocampus and basal ganglia can be differently engaged relative to, e.g. BA 44, at different stages of syntactic learning [76].

**Figure 3.**
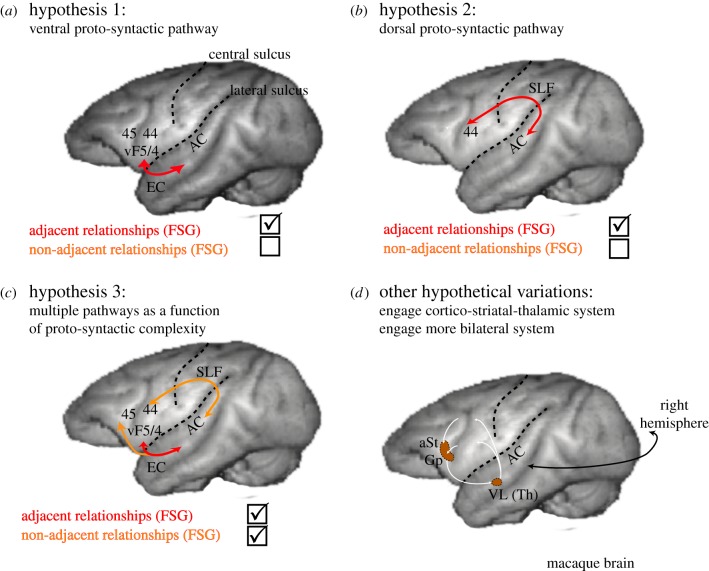
Hypothetical proto-syntactic learning capabilities and neurobiological substrates in monkeys. (*a*) Hypothesis 1 illustrates a ventral pathway linking the supratemporal plane with inferior frontal cortex. Here, the animals are only able to learn adjacent relationships in finite-state grammars (FSGs). (*b*) Hypothesis 2 illustrates a dorsal pathway supporting the learning of FSG. (*c*) Hypothesis 3 illustrates the reliance on multiple pathways and regions depending on the complexity of the FSG patterns that can be learned (e.g. for adjacent relationships, a ventral pathway; for non-adjacent relationships, a dorsal pathway and/or a different part of the ventral pathway). (*d*) A discussion of variations to these hypotheses, see text. AC, auditory cortex; aSt, anterior striatum; Gp, globus pallidus; vF4/vF5, ventral frontal cortical areas F4 and F5 [82]; VL, ventro-lateral thalamus; 44/45, Brodmann areas 44/45.

Based on these considerations, several hypotheses can be articulated that consider the level of complexity that non-human primates are capable of learning and the neurobiological regions and pathways that might be engaged. For clarity in illustration, in figure 3 we subdivide the likely AGL capabilities into abilities for evaluating adjacent relationships alone or with non-adjacent relationships. See §2 and figure 1 for other aspects of syntactic complexity that could also be useful for testing. Moreover, since we are considering AGL of the temporal structure of sensory elements, it is an open question whether, all things equal, all presumed homologues of the pathways that have been described in humans would be engaged (e.g. dorsal pathway I to premotor cortex that is engaged in sensory-to-motor mapping might not be involved in this case). Also, although traditionally the dorsal arcuate fasciculus is considered as the classical language pathway linking Broca's and Wernicke's territories, the ventral pathway(s) and their role in language processes are being emphasized by some groups [77–79]. However, although the ventral UF and EC pathways are anatomically evident in non-human primates, our hypotheses at this point only make predictions about the EC pathway since it is the primary ventral fronto-temporal tract that can currently be resolved with *in vivo* connectivity studies of the IFG in monkeys and apes [80,81].

### (a) Hypothesis 1: ventral pathway for proto-syntactic learning

There is evidence that tamarin monkeys are able to learn adjacent relationships in FSGs, but are insensitive to violations of more complex grammatical patterns [11]. Also, human fMRI results suggest that the processing of such adjacent relationships engages the FOP, more so than Broca's territory [50]. However, in humans the processing of various sorts of non-adjacent relationships in artificial grammars [13,50], including those with hierarchical structure [43], engages at least Broca's territory, e.g. BA 44. Thus, one hypothesis is that the involvement of Broca's territory (and the dorsal SLF pathway) would not be seen in non-human primates [9,50], especially if the animals are not capable of evaluating *non-adjacent relationships*. In this scenario, when evaluating adjacent relationships or simpler syntactic-related relationships, both humans and some species of non-human primates might engage a ventral pathway (EC and/or UF) interconnecting anterior temporal lobe regions and frontal cortical areas that are inferior to BA 44/45 (e.g. in monkeys, the frontal opercular areas or areas vF5/F4 [82]). We illustrate this scenario in figure 3*a* (hypothesis 1: ventral pathway). If, the non-human primates are able to evaluate *non-adjacent relationships* and for this engage the ventral pathway, then this would suggest that the human dorsal pathway involving the arcuate fasciculus differentiated during language evolution to support increasing syntactic complexity, as Rilling *et al*. [80] have suggested.

### (b) Hypothesis 2: dorsal pathway for proto-syntactic learning

A second hypothesis is that the processing of FSGs with only *adjacent relationships* engages monkey homologues of BA 44/45 and the dorsal SLF pathway, as illustrated in figure 3*b* (hypothesis 2: dorsal pathway). This would also suggest that the dorsal pathway differentiated after the split from a common ancestor to support the learning of greater syntactic complexity in humans.

### (c) Hypothesis 3: multiple pathways in non-human primates for proto-syntactic learning depend on syntactic complexity

A third hypothesis is that different brain regions and pathways are engaged depending on the complexity of the grammars that can be learned. For instance, any combination of the following might be possible: (i) parts of the ventral pathway linking temporal lobe regions to monkey homologues of the human FOP are engaged in the processing of adjacent relationships in FSGs; (ii) the dorsal pathway is relied on for processing greater complexity in FSGs, such as non-adjacent relationships [13]; and/or (iii) different parts of the ventral pathway are engaged in evaluating either adjacent or non-adjacent relationships (figure 3*c*: hypothesis 3: multiple pathways). The combination of these scenarios in monkeys might be viewed to be the most comparable to how the human brain processes syntactic complexity, but there could be subtle differences. For instance, would the processing of comparable adjacent and non-adjacent relationships in artificial grammars engage a broader set of regions in frontal cortex in monkeys? If so, this could suggest a different form of functional differentiation during human language evolution from the ones considered for the other hypotheses above. For example, the ventral pathway and BA 45 might in humans have had to differentiate to support the combination of semantic and syntactic relationships [42].

### (d) Other variants and hypotheses

The human syntactic learning network is also not entirely left lateralized [70], nor is the processing of communication sounds in humans, chimpanzees or monkeys [62,63]. Thus, it is possible that the right hemisphere in non-human primates might show some of the homotopic regions and connectivity illustrated here for the left hemisphere. Also, for brevity, the hypotheses of figure 3 do not illustrate the possible greater or lesser reliance on subcortical structures (such as the striatum and basal ganglia) or cerebellum to support, for instance, the implicit learning of artificial-grammar sequences. Fitch [83] proposed three interesting hypotheses regarding how the human syntactic network might differ from ancestral variants present in living non-human animals. First is the notion that human vocal learning involves a direct pathway between the regions required for vocal learning and the laryngeal motoneurons in the nucleus ambiguus in the brainstem. As suggested in §3 above, we would not expect the vocal production pathway to be engaged in (at least) the implicit learning of artificial-grammar sequences in non-human primates; for more details, see Petkov & Jarvis [25]. The second Fitch hypothesis regarding the specialization of the arcuate fasciculus [80] is considered in detail above. The third of the hypotheses considers the architectonic and other specializations of Broca's territory, e.g. BA 44, which, if present, might be evident in differences in the neurobiological activity and/or connectivity patterns between humans and monkeys in relation to their behavioural capabilities.

In summary, it is possible that humans engage at least Broca's territory and a dorsal pathway to process grammatical complexity in a way that may not be evident in non-human primates (hypothesis 1 in figure 3). Other possibilities are that monkeys may engage homologues of BA 44 and parts of the dorsal SLF tract for grammars perceived as simple by humans (hypothesis 2), or that there is a general correspondence between how human and monkey brain networks evaluate artificial-grammar complexity (hypothesis 3) with more or less subtle differences in hemispheric lateralization and/or cortical and subcortical engagement. It remains to be seen how a proto-syntactic network in monkeys would compare to the network humans that subserves syntactic learning.

## 5. Conclusions

At least conceptually, the approach with non-human primates and possibly also the one that might be taken with other so-called ‘vocal non-learning’ animals must differ from the approaches that are being taken with vocal learning animals, such as songbirds. On the other hand, the comparative testing of behaviour and neurobiology needs to be done as similarly as possible across the species so that data can be compared. We have aimed to build on the efforts of the international scientific community to understand the origins of language and to open new pathways for pursuing language homologues in non-human animals that tend to be dismissed from consideration. Work has also begun to refine the comparative behavioural testing of humans and non-human animals on AGL paradigms and we have begun to obtain initial results on monkey AGL with fMRI in our laboratory [84]. The constraints that are imposed by working with animals that are limited vocal learners can also be positively viewed as providing important insights, guidance and predictions into the ancestral state of the human language-related network and its generic processing capabilities. Thereby, the comparative approach remains important for understanding language evolution and for the development of useful animal model systems to study the evolutionarily conserved aspects of the human language-related network at the cellular and molecular levels.

## Endnote

1 In some cases, neuronal studies in humans are possible [16]. However, great care is required for interpreting the results from clinical patients that either involve or neighbour pathological regions that are being monitored for neurosurgical resection. In all cases, information from cell and molecular studies in animals can enhance the data from neuronal-level study in humans, provided that the level of correspondence across the species is tested using a common bridging technique.

## References

[1] Hurford J. R. 2012 The origins of grammar: language in the light of evolution. Oxford, UK: Oxford University Press

[2] Tallerman M. 2011 What is syntax? In The Oxford handbook of language evolution (eds Tallerman M. Gibson K. R.), pp. 442–455 Oxford, UK: Oxford University Press

[3] Fitch W. T. Friederici A. D. 2012 Artificial grammar learning meets formal language theory: an overview. Phil. Trans. R. Soc. B 367, 1933–1955 10.1098/rstb.2012.0103 (doi:10.1098/rstb.2012.0103) PMC3367694 22688631

[4] Jäger G. Rogers J. 2012 Formal language theory: refining the Chomsky hierarchy. Phil. Trans. R. Soc. B 367, 1956–1970 10.1098/rstb.2012.0077 (doi:10.1098/rstb.2012.0077) PMC3367686 22688632

[5] Bickerton D. Szathmary E. 2009 Biological foundations and origin of syntax. Cambridge, MA: MIT Press

[6] Berwick R. C. Okanoya K. Beckers G. J. Bolhuis J. J. 2011 Songs to syntax: the linguistics of birdsong. Trends Cogn. Sci. 15, 113–121 10.1016/j.tics.2011.01.002 (doi:10.1016/j.tics.2011.01.002) 21296608

[7] Hauser M. D. Chomsky N. Fitch W. T. 2002 The faculty of language: what is it, who has it, and how did it evolve? Science 298, 1569–1579 10.1126/science.298.5598.1569 (doi:10.1126/science.298.5598.1569) 12446899

[8] Fitch W. T. 2000 The evolution of speech: a comparative review. Trends Cogn. Sci. 4, 258–267 10.1016/S1364-6613(00)01494-7 (doi:10.1016/S1364-6613(00)01494-7) 10859570

[9] Friederici A. D. 2004 Processing local transitions versus long-distance syntactic hierarchies. Trends Cogn. Sci. 8, 245–247 10.1016/j.tics.2004.04.013 (doi:10.1016/j.tics.2004.04.013) 15165545

[10] Reber A. S. 1967 Implicit learning of artificial grammars. J. Verbal Learn. Verbal Behav. 6, 855–863 10.1016/S0022-5371(67)80149-X (doi:10.1016/S0022-5371(67)80149-X)

[11] Fitch W. T. Hauser M. D. 2004 Computational constraints on syntactic processing in a nonhuman primate. Science 303, 377–380 10.1126/science.10894013 (doi:10.1126/science.10894013) 14726592

[12] Gentner T. Q. Fenn K. M. Margoliash D. Nusbaum H. C. 2006 Recursive syntactic pattern learning by songbirds. Nature 440, 1204–1207 10.1038/nature04675 (doi:10.1038/nature04675) 16641998 PMC2653278

[13] Petersson K. M. Folia V. Hagoort P. 2010 What artificial grammar learning reveals about the neurobiology of syntax. Brain Lang. 120, 83–95 10.1016/j.bandl.2010.08.003 (doi:10.1016/j.bandl.2010.08.003) 20943261

[14] Marcus G. F. Vijayan S. Rao S. B. Vishton P. M. 1999 Rule learning by seven-month-old infants. Science 283, 77–80 10.1126/science.283.5398.77 (doi:10.1126/science.283.5398.77) 9872745

[15] Bolhuis J. J. Okanoya K. Scharff C. 2010 Twitter evolution: converging mechanisms in birdsong and human speech. Nat. Rev. Neurosci. 11, 747–759 10.1038/nrn2931 (doi:10.1038/nrn2931) 20959859

[16] Sahin N. T. Pinker S. Cash S. S. Schomer D. Halgren E. 2009 Sequential processing of lexical, grammatical, and phonological information within Broca's area. Science 326, 445–449 10.1126/science.1174481 (doi:10.1126/science.1174481) 19833971 PMC4030760

[17] Chomsky N. 1957 Syntactic structures. The Hague, The Netherlands: Mouton

[18] Noad M. J. Cato D. H. Bryden M. M. Jenner M. N. Jenner K. C. 2000 Cultural revolution in whale songs. Nature 408, 537 10.1038/35046199 (doi:10.1038/35046199) 11117730

[19] Jarvis E. D. 2004 Learned birdsong and the neurobiology of human language. Ann. NY Acad. Sci. 1016, 749–777 10.1196/annals.1298.038 (doi:10.1196/annals.1298.038) 15313804 PMC2485240

[20] Pepperberg I. M. 2010 Vocal learning in grey parrots: a brief review of perception, production, and cross-species comparisons. Brain Lang. 115, 81–91 10.1016/j.bandl.2009.11.002 (doi:10.1016/j.bandl.2009.11.002) 20199805

[21] Boughman J. W. 1998 Vocal learning by greater spear-nosed bats. Proc. R. Soc. Lond. B 265, 227–233 10.1098/rspb.1998.0286 (doi:10.1098/rspb.1998.0286) PMC1688873 9493408

[22] Janik V. M. Slater P. J. 1997 Vocal learning in mammals. Adv. Study Behav. 26, 59–99 10.1016/S0065-3454(08)60377-0 (doi:10.1016/S0065-3454(08)60377-0)

[23] Marler P. 2000 Origin of music and speech: insights from animals. In The origins of music (eds Wallin N. L. Merker B. Brown S.), pp. 31–50 Cambridge, MA: Massachusetts Institute of Technology

[24] Snowdon C. T. 2009 Plasticity of communication in nonhuman primates. In Advances in the study of behavior (eds Naguib M. Janik V. M.), pp. 239–276 Burlington, VT: Academic Press

[25] Petkov C. I. Jarvis E. In press Birds, primates and spoken language origins: behavioral phenotypes and neurobiological substrates. Front. Evol. Neurosci. 10.3389/fnevo.2012.00012 PMC3419981 22912615

[26] Ouattara K. Lemasson A. Zuberbuhler K. 2009 Campbell's monkeys concatenate vocalizations into context-specific call sequences. Proc. Natl Acad. Sci. USA 106, 22 026–22 031 10.1073/pnas.0908118106 (doi:10.1073/pnas.0908118106) PMC2799830 20007377

[27] Arnold K. Zuberbuhler K. 2006 Language evolution: semantic combinations in primate calls. Nature 441, 303 10.1038/441303a (doi:10.1038/441303a) 16710411

[28] Marcus G. F. Vijayan S. Bandi Rao S. Vishton P. M. 1999 Rule learning by seven-month-old infants. Science 283, 77–80 10.1126/science.283.5398.77 (doi:10.1126/science.283.5398.77) 9872745

[29] Moore B. R. 2004 The evolution of learning. Biol. Rev. Camb. Philos. Soc. 79, 301–335 10.1017/S1464793103006225 (doi:10.1017/S1464793103006225) 15191226

[30] Joshi A. K. 1985 Tree-adjoining grammars: how much context sensitivity is required to provide reasonable structural descriptions? In Natural language parsing (eds Dowty D. Karttunen L. Zwicky A.), pp. 206–250 Cambridge, UK: Cambridge University Press

[31] Fitch W. T. Friederici A. D. Hagoort P. 2012 Pattern perception and computational complexity: introduction to the special issue. Phil. Trans. R. Soc. B 367, 1925–1932 10.1098/rstb.2012.0099 (doi:10.1098/rstb.2012.0099) PMC3367691 22688630

[32] Abe K. Watanabe D. 2011 Songbirds possess the spontaneous ability to discriminate syntactic rules. Nat. Neurosci. 14, 1067–1074 10.1038/nn.2869nn.2869 (doi:10.1038/nn.2869nn.2869) 21706017

[33] van Heijningen C. A. de Visser J. Zuidema W. ten Cate C. 2009 Simple rules can explain discrimination of putative recursive syntactic structures by a songbird species. Proc. Natl Acad. Sci. USA 106, 20 538–20 543 10.1073/pnas.0908113106 (doi:10.1073/pnas.0908113106) PMC2787117 19918074

[34] ten Cate C. Okanoya K. 2012 Revisiting the syntactic abilities of non-human animals: natural vocalizations and artificial grammar learning. Phil. Trans. R. Soc. B 367, 1984–1994 10.1098/rstb.2012.0055 (doi:10.1098/rstb.2012.0055) PMC3367684 22688634

[35] Teal T. Taylor C. E. (eds) 2000 Effects of compression on language evolution. In Artificial life, pp. 129–143 Cambridge, MA: MIT Press 10.1162/106454600568366 10953250

[36] Okanoya K. 2004 The Bengalese finch: a window on the behavioral neurobiology of birdsong syntax. Ann. NY Acad. Sci. 1016, 724–735 10.1196/annals.1298.0261016/1/724 (doi:10.1196/annals.1298.0261016/1/724) 15313802

[37] Honda E. Okanoya K. 1999 Acoustical and syntactical comparisons between songs of the white-backed munia (*Lonchura striata*) and its domesticated strain, the Bengalese finch (*Lonchura striata* var. domestica). Zool. Sci. 16, 319–326 10.2108/zsj.16.319 (doi:10.2108/zsj.16.319) 22927235

[38] Folia V. Forkstam C. Ingvar M. Hagoort P. Petersson K. M. 2011 Implicit artificial syntax processing: genes, preference, and bounded recursion. Biolinguistics 5, 105–132

[39] Udden J. Ingvar M. Hagoort P. Petersson K. M. In press Implicit acquisition of grammars with crossed and nested non-adjacent dependencies: investigating the push-down stack model. Cogn. Sci. (doi:10.1111/j.1551-6709.2012.01235.x) 10.1111/j.1551-6709.2012.01235.x 22452530

[40] Friederici A. D. Mueller J. L. Oberecker R. 2011 Precursors to natural grammar learning: preliminary evidence from 4-month-old infants. PLoS ONE 6, e17920 10.1371/journal.pone.0017920 (doi:10.1371/journal.pone.0017920) 21445341 PMC3062547

[41] Perani D. Saccuman M. C. Scifo P. Awander A. Spada D. Baldoli C. Poloniato A. Lohmann G. Friederici A. D. 2011 Neural language networks at birth. Proc. Natl Acad. Sci. USA 108, 16 056–16 061 10.1073/pnas.1102991108 (doi:10.1073/pnas.1102991108) PMC3179044 21896765

[42] Friederici A. D. 2011 The brain basis of language processing: from structure to function. Physiol. Rev. 91, 1357–1392 10.1152/physrev.00006.2011 (doi:10.1152/physrev.00006.2011) 22013214

[43] Bahlmann J. Schubotz R. I. Friederici A. D. 2008 Hierarchical artificial grammar processing engages Broca's area. Neuroimage 42, 525–534 10.1016/j.neuroimage.2008.04.249 (doi:10.1016/j.neuroimage.2008.04.249) 18554927

[44] Saffran J. Hauser M. Seibel R. Kapfhamer J. Tsao F. Cushman F. 2008 Grammatical pattern learning by human infants and cotton-top tamarin monkeys. Cognition 107, 479–500 10.1016/j.cognition.2007.10.010 (doi:10.1016/j.cognition.2007.10.010) 18082676 PMC2386981

[45] Saffran J. R. Johnson E. K. Aslin R. N. Newport E. L. 1999 Statistical learning of tone sequences by human infants and adults. Cognition 70, 27–52 10.1016/S0010-0277(98)00075-4 (doi:10.1016/S0010-0277(98)00075-4) 10193055

[46] Murphy R. A. Mondragon E. Murphy V. A. 2008 Rule learning by rats. Science 319, 1849–1851 10.1126/science.1151564 (doi:10.1126/science.1151564) 18369151

[47] Kirby S. Cornish H. Smith K. 2008 Cumulative cultural evolution in the laboratory: an experimental approach to the origins of structure in human language. Proc. Natl Acad. Sci. USA 105, 10 681–10 686 10.1073/pnas.0707835105 (doi:10.1073/pnas.0707835105) PMC2504810 18667697

[48] Saffran J. R. Aslin R. N. Newport E. L. 1996 Statistical learning by 8-month-old infants. Science 274, 1926–1928 10.1126/science.274.5294.1926 (doi:10.1126/science.274.5294.1926) 8943209

[49] Stobbe N. Westphal-Fitch G. Aust U. Fitch W. T. 2012 Visual artificial grammar learning: comparative research on humans, kea (*Nestor notabilis*) and pigeons (*Columba livia*). Phil. Trans. R. Soc. B 367, 1995–2006 10.1098/rstb.2012.0096 (doi:10.1098/rstb.2012.0096) PMC3367688 22688635

[50] Friederici A. D. Bahlmann J. Heim S. Schubotz R. I. Anwander A. 2006 The brain differentiates human and non-human grammars: functional localization and structural connectivity. Proc. Natl Acad. Sci. USA 103, 2458–2463 10.1073/pnas.0509389103 (doi:10.1073/pnas.0509389103) 16461904 PMC1413709

[51] Petersson K. M. Forkstam C. Ingvar M. 2004 Artificial syntactic violations activate Broca's region. Cogn. Sci. 28, 383–407 10.1016/j.cogsci.2003.12.003 (doi:10.1016/j.cogsci.2003.12.003)

[52] Squire L. R. Zola S. M. 1996 Structure and function of declarative and nondeclarative memory systems. Proc. Natl Acad. Sci. USA 93, 13 515–13 522 10.1073/pnas.93.24.13515 (doi:10.1073/pnas.93.24.13515) 8942965 PMC33639

[53] Logothetis N. K. Guggenberger H. Peled S. Pauls J. 1999 Functional imaging of the monkey brain. Nat. Neurosci. 2, 555–562 10.1038/9210 (doi:10.1038/9210) 10448221

[54] Logothetis N. K. 2008 What we can do and what we cannot do with fMRI. Nature 453, 869–878 10.1038/nature06976 (doi:10.1038/nature06976) 18548064

[55] Petkov C. I. Kayser C. Augath M. Logothetis N. K. 2006 Functional imaging reveals numerous fields in the monkey auditory cortex. PLoS Biol. 4, e215 10.1371/journal.pbio.0040215 (doi:10.1371/journal.pbio.0040215) 16774452 PMC1479693

[56] Baumann S. Griffiths T. D. Sun L. Petkov C. I. Thiele A. Rees A. 2011 Orthogonal representation of sound dimensions in the primate midbrain. Nat. Neurosci. 14, 423–425 10.1038/nn.2771 (doi:10.1038/nn.2771) 21378972 PMC3068195

[57] Petkov C. I. Kayser C. Steudel T. Whittingstall K. Augath M. Logothetis N. K. 2008 A voice region in the monkey brain. Nat. Neurosci. 11, 367–374 10.1038/nn2043 (doi:10.1038/nn2043) 18264095

[58] Poremba A. Malloy M. Saunders R. C. Carson R. E. Herscovitch P. Mishkin M. 2004 Species-specific calls evoke asymmetric activity in the monkey's temporal poles. Nature 427, 448–451 10.1038/nature02268 (doi:10.1038/nature02268) 14749833

[59] Gil-da-Costa R. Braun A. Lopes M. Hauser M. D. Carson R. E. Herscovitch P. Martin A. 2004 Toward an evolutionary perspective on conceptual representation: species-specific calls activate visual and affective processing systems in the macaque. Proc. Natl Acad. Sci. USA 101, 17 516–17 521 10.1073/pnas.0408077101 (doi:10.1073/pnas.0408077101) PMC536037 15583132

[60] Joly O. Ramus F. Pressnitzer D. Vanduffel W. Orban G. A. 2011 Interhemispheric differences in auditory processing revealed by fMRI in awake rhesus monkeys. Cereb. Cortex 22, 838–854 10.1093/cercor/bhr150 (doi:10.1093/cercor/bhr150) 21709178

[61] Taglialatela J. P. Russell J. L. Schaeffer J. A. Hopkins W. D. 2009 Visualizing vocal perception in the chimpanzee brain. Cereb. Cortex 19, 1151–1157 10.1093/cercor/bhn157 (doi:10.1093/cercor/bhn157) 18787228 PMC2665158

[62] Petkov C. I. Logothetis N. K. Obleser J. 2009 Where are the human speech and voice regions and do other animals have anything like them? Neuroscientist 15, 419–429 10.1177/1073858408326430 (doi:10.1177/1073858408326430) 19516047

[63] Wilson B. Petkov C. 2011 Communication and the primate brain: insights from neuroimaging studies in humans, chimpanzees and macaques. Hum. Biol. 83, 175–189 10.3378/027.083.0203 (doi:10.3378/027.083.0203) 21615285 PMC3398142

[64] Rauschecker J. P. Tian B. Hauser M. 1995 Processing of complex sounds in the macaque nonprimary auditory cortex. Science 268, 111–114 10.1126/science.7701330 (doi:10.1126/science.7701330) 7701330

[65] Russ B. E. Ackelson A. L. Baker A. E. Cohen Y. E. 2008 Coding of auditory-stimulus identity in the auditory non-spatial processing stream. J. Neurophysiol. 99, 87–95 10.1152/jn.01069.2007 (doi:10.1152/jn.01069.2007) 18003874 PMC4091985

[66] Romanski L. M. Averbeck B. B. Diltz M. 2005 Neural representation of vocalizations in the primate ventrolateral prefrontal cortex. J. Neurophysiol. 93, 734–747 10.1152/jn.00675.2004 (doi:10.1152/jn.00675.2004) 15371495

[67] Belin P. Zatorre R. J. Lafaille P. Ahad P. Pike B. 2000 Voice-selective areas in human auditory cortex. Nature 403, 309–312 10.1038/35002078 (doi:10.1038/35002078) 10659849

[68] von Kriegstein K. Eger E. Kleinschmidt A. Giraud A. L. 2003 Modulation of neural responses to speech by directing attention to voices or verbal content. Brain Res. Cogn. Brain Res. 17, 48–55 10.1016/S0926-6410(03)00079-X (doi:10.1016/S0926-6410(03)00079-X) 12763191

[69] Perrodin C. Kayser C. Logothetis N. K. Petkov C. I. 2011 Voice cells in the primate temporal lobe. Curr. Biol. 21, 1408–1415 10.1016/j.cub.2011.07.028 (doi:10.1016/j.cub.2011.07.028) 21835625 PMC3398143

[70] Hagoort P. 2009 Reflections on the neurobiology of syntax. In Biological foundations and origins of syntax (eds. Bickerton D. Szathmary E.), pp. 279–298 Cambridge, MA: MIT Press

[71] Tyler L. K. Marslen-Wilson W. D. Randall B. Wright P. Devereux B. J. Zhuang J. Papoutsi M. Stamatakis E. A. 2011 Left inferior frontal cortex and syntax: function, structure and behaviour in patients with left hemisphere damage. Brain 134, 415–431 10.1093/brain/awq369 (doi:10.1093/brain/awq369) 21278407 PMC3030769

[72] Marslen-Wilson W. D. Tyler L. K. 2007 Morphology, language and the brain: the decompositional substrate for language comprehension. Phil. Trans. R. Soc. B 362, 823–836 10.1098/rstb.2007.2091 (doi:10.1098/rstb.2007.2091) 17395577 PMC2430000

[73] Rauschecker J. P. Scott S. K. 2009 Maps and streams in the auditory cortex: nonhuman primates illuminate human speech processing. Nat. Neurosci. 12, 718–724 10.1038/nn.2331 (doi:10.1038/nn.2331) 19471271 PMC2846110

[74] Hickok G. Poeppel D. 2007 The cortical organization of speech processing. Nat. Rev. Neurosci. 8, 393–402 10.1038/nrn2113 (doi:10.1038/nrn2113) 17431404

[75] Tyler L. K. Wright P. Randall B. Marslen-Wilson W. D. Stamatakis E. A. 2010 Reorganization of syntactic processing following left-hemisphere brain damage: does right-hemisphere activity preserve function? Brain 133, 3396–3408 10.1093/brain/awq262 (doi:10.1093/brain/awq262) 20870779 PMC2965424

[76] Opitz B. Friederici A. D. 2003 Interactions of the hippocampal system and the prefrontal cortex in learning language-like rules. Neuroimage 19, 1730–1737 10.1016/S1053-8119(03)00170-8 (doi:10.1016/S1053-8119(03)00170-8) 12948727

[77] Friederici A. D. 2009 Pathways to language: fiber tracts in the human brain. Trends Cogn. Sci. 13, 175–181 10.1016/j.tics.2009.01.001 (doi:10.1016/j.tics.2009.01.001) 19223226

[78] Weiller C. Musso M. Rijntjes M. Saur D. 2009 Please don't underestimate the ventral pathway in language. Trends Cogn. Sci. 13, 369–370 10.1016/j.tics.2009.06.007 (doi:10.1016/j.tics.2009.06.007) 19716753

[79] Papoutsi M. Stamatakis E. A. Griffiths J. Marslen-Wilson W. D. Tyler L. K. 2011 Is left fronto-temporal connectivity essential for syntax? Effective connectivity, tractography and performance in left-hemisphere damaged patients. Neuroimage 58, 656–664 10.1016/j.neuroimage.2011.06.036 (doi:10.1016/j.neuroimage.2011.06.036) 21722742

[80] Rilling J. K. Glasser M. F. Preuss T. M. Ma X. Zhao T. Hu X. Behrens T. E. 2008 The evolution of the arcuate fasciculus revealed with comparative DTI. Nat. Neurosci. 11, 426–428 10.1038/nn2072 (doi:10.1038/nn2072) 18344993

[81] Petrides M. Pandya D. N. 2009 Distinct parietal and temporal pathways to the homologues of Broca's area in the monkey. PLoS Biol. 7, e1000170 10.1371/journal.pbio.1000170 (doi:10.1371/journal.pbio.1000170) 19668354 PMC2714989

[82] Saleem K. S. Logothetis N. K. 2007 A combined MRI and histology: atlas of the rhesus monkey brain in stereotaxic coordinates. London, UK: Academic Press

[83] Fitch W. T. 2012 The evolution of syntax: an exaptationist perspective. Front. Evol. Neurosci. 3, 9 10.3389/fnevo.2011.00009 (doi:10.3389/fnevo.2011.00009) 22207847 PMC3245538

[84] Wilson B. Collison M. G. Slater H. Smith K. Marslen-Wilson W. Petkov C. 2011 Behaviour and functional imaging of ‘artificial-grammar’ sequence learning in Rhesus macaques. In Proc. Neuroscience 2011, Washington DC, 12–16 November 2011. No. 173.18. Society for Neuroscience.

